# Pitch Processing in Children with Williams Syndrome: Relationships between Music and Prosody Skills

**DOI:** 10.3390/brainsci4020376

**Published:** 2014-05-15

**Authors:** Pastora Martínez-Castilla, María Sotillo

**Affiliations:** 1 Department of Developmental and Educational Psychology, National Distance Education University (UNED), C/Juan del Rosal, n°10, 28040 Madrid, Spain; 2 Department of Basic Psychology, Autonomous University of Madrid, C/Iván Paulov, n°6, 28049 Madrid, Spain; E-Mail: maria.sotillo@uam.es

**Keywords:** Williams syndrome, pitch processing, music, prosody, modularity, relationships

## Abstract

Williams syndrome (WS), a genetic neurodevelopmental disorder, has been taken as evidence that music and language constitute separate modules. This research focused on the linguistic component of prosody and aimed to assess whether relationships exist between the pitch processing mechanisms for music and prosody in WS. Children with WS and typically developing individuals were presented with a musical pitch and two prosody discrimination tasks. In the musical pitch discrimination task, participants were required to distinguish whether two musical tones were the same or different. The prosody discrimination tasks evaluated participants’ skills for discriminating pairs of prosodic contours based on pitch or pitch, loudness and length, jointly. In WS, musical pitch discrimination was significantly correlated with performance on the prosody task assessing the discrimination of prosodic contours based on pitch only. Furthermore, musical pitch discrimination skills predicted performance on the prosody task based on pitch, and this relationship was not better explained by chronological age, vocabulary or auditory memory. These results suggest that children with WS process pitch in music and prosody through shared mechanisms. We discuss the implications of these results for theories of cognitive modularity. The implications of these results for intervention programs for individuals with WS are also discussed.

## 1. Introduction

The study of the relationships between music and language arouses substantial interest because of its potential to unravel issues regarding the modularity of the two domains [1]. The debate remains open: data suggesting that music and language are dissociated and constitute different modules (e.g., [2,3]) coexist with evidence for the existence of shared processing mechanisms (e.g., [4,5,6]; for a review, see [7]). Yet, when referring to prosody, *i.e.*, the melodic and temporal properties of language, even the theoretical frames claiming modularity admit that there is some overlap between the mechanisms for processing prosody and music [8]. The existence of shared mechanisms between music and prosody processing may be accounted for by the fact that both music and prosody are expressed through variations in pitch, loudness and length (duration), *i.e.*, through the same parameters (e.g., [9,10,11,12]). Among these parameters, pitch is considered to be especially important for revealing the modularity or possible relationships in music and prosody processing because of its important role for conveying information in both domains [13]. Given that domain-specificity is essential for assuming modularity [14], if music and prosody were completely modular, the two domains would be subserved by different mechanisms for processing pitch. Instead, as we shall see, in typically developing (TD) individuals, the parameter of pitch seems to be processed in music and prosody through common cognitive and neural mechanisms [15,16,17,18,19,20,21,22,23,24,25,26,27]. Although the processing of pitch seems not to be specific to the domain of music, but to be shared with prosody in TD individuals, this could not occur in individuals with neurodevelopmental disorders, in whom the cognitive architecture is likely to be atypical [28]. Such is the case of Williams syndrome (WS), a disorder that, despite its genetic, neurobiological and cognitive abnormalities, has been taken as evidence of the existence of separate modules for language and for music [29,30,31] and that has triggered controversy regarding the modularity of mind (e.g., [28,31,32]). In this paper, we evaluate whether individuals with WS process pitch in music and prosody through common cognitive mechanisms. Our study may therefore contribute to the theoretical debates regarding modularity in WS and, more specifically, regarding the modularity of music and the language component of prosody.

In the following subsections, we first briefly address the views on modularity for language and music in WS. We then consider the research on pitch processing in music and prosody in this population and present a short review of the literature on the relationships between music and prosody for pitch processing in TD individuals. Finally, the aims and hypotheses of the study are presented.

### 1.1. The Debate on the Modularity of Mind for Language and Music in Williams Syndrome

WS is a neurodevelopmental disorder of genetic origin that is caused by a hemizygous deletion in 7q11.23 [33]. It occurs in 1:20,000 to 7500 live births and is thus a rare syndrome [34,35]. In addition to mild to severe intellectual disability, individuals with WS present an uneven cognitive profile (e.g., [36,37]). Early descriptions of this cognitive profile highlighted how individuals with WS seemed to have good language skills together with intellectual disability and severe impairments for visuo-spatial cognition (e.g., [38,39]). The early descriptions led to claiming that language is independent of other cognitive systems; consequently, WS was considered to illustrate theories of cognitive modularity (e.g., [31]). Similarly, music skills have been traditionally described as outstanding and preserved, and this view of music skills has also been used to support theories of cognitive modularity (e.g., [29,30,40]).

At present, a large body of research shows that neither language nor music can be considered intact in WS (e.g., [41,42,43]). Instead, both language and music seem to be characterized by a complex pattern of strengths and weaknesses (e.g., [44,45]). Furthermore, unlike the initial claims that language and music are different modules independent of other cognitive systems [30,31], increasing evidence supports the opposite conclusion, and WS is argued to be able to illustrate the interdependence of different cognitive domains [46]. Thus, contrasting with early hypotheses about the dissociation between language and visuo-spatial skills as evidence of the modularity of the two domains, the deficits found in spatial language suggest that the two domains are connected [47,48]. In music, recent studies have shown that the rhythm skills of individuals with WS are affected by their cognitive deficits [49]. These findings suggest that music is indeed related to other cognitive processes in WS [49]. When studied together, positive correlations have been found between music (e.g., pitch discrimination) and simple measures of language (e.g., receptive vocabulary and auditory closure) in individuals with WS [50]. Far from the views of cognitive modularity, these correlations have been interpreted as evidence for common basic auditory processing mechanisms between the two domains in WS [50].

Although WS has often been used to provide evidence to support theories of cognitive modularity in TD individuals, neuroconstructivist approaches argue that neurodevelopmental disorders should not be used to elucidate the cognitive architecture of TD individuals, *i.e.*, it should not be assumed that neurodevelopmental disorders can show how the cognitive architecture is organized in TD individuals [51]. From this view, neurodevelopmental disorders may show not only developmental pathways that differ from those found in TD individuals, but also atypical cognitive processes that underlie behavior (e.g., [28,32]). Thus, studies on neurodevelopmental disorders, such as those on WS, apart from being interesting in themselves, have the potential to challenge theoretical models of cognition (e.g., [52]).

### 1.2. Pitch Processing in Music and Prosody in Individuals with Williams Syndrome

As mentioned above, initially, it was claimed that both music and prosody, as a component of language, are preserved in WS [29,30,40,53,54,55,56]. In the field of music, the first studies on pitch processing were crucial in supporting this view. Thus, early studies on absolute pitch, *i.e.*, a skill consisting in being able to identify a musical pitch without a reference tone [57], argued that individuals with WS present excellent abilities in this regard [29,40]. While absolute pitch is described as being a rare skill in TD individuals, it has been claimed that the incidence of this music skill is higher in individuals with WS [29,40]. Moreover, the fact that this music skill seemed to be excellent despite the intellectual disability that is characteristic of individuals with WS was interpreted as evidence of cognitive modularity [29,40]. Nevertheless, recent studies have brought into question these claims. Thus, it has been reported that individuals with WS do not have remarkable abilities for absolute pitch and that, as in the TD population, this skill is also rare in WS [58].

Furthermore, musical pitch processing not only is unremarkable, but also seems to be impaired in individuals with WS. When presented with pairs of musical tones to be discriminated, children, adolescents and adults with WS perform significantly worse than their TD peers matched on chronological age [45,59]. Similar results have been found in more complex tasks, such as chord analysis or tonal memory [59]. Moreover, results with respect to tasks for which participants have to discriminate pairs of melodies changing in their constituent notes suggest that the incidence of amusia (*i.e.*, a marked impairment in pitch perception, also called tone-deafness) may be higher in WS than in the TD population [60]. Apart from the deficits reported, atypical processes underlying musical pitch processing have been found in individuals with WS. Thus, unlike TD individuals, those with WS seem to present a lack of sensitivity to pitch contour clues in melodies [42,61].

With respect to prosody, a body of studies has also shown that, unlike what was initially suggested, the prosody skills of individuals with WS, including those related to pitch processing, are impaired [62,63,64,65,66]. Thus, children, adolescents and adults with WS perform worse than their TD peers of the same chronological age when they are required to discriminate pairs of prosodic contours based on pitch [62,66]. Similar results have been found when other parameters (e.g., length and loudness) are also involved in the prosodic contours to be discriminated [62,66]. Individuals with WS have difficulty not only discriminating pitch in prosodic contours, but also understanding the meaning expressed by this parameter in speech prosody [62,63,64,65,66].

Despite the importance of studying the relationships in the processing of pitch between music and prosody in individuals with WS, no prior study has tackled this issue and, thus, to date, whether individuals with WS process pitch in music and prosody through common mechanisms remains unknown. Instead, as shown below, an increasing body of studies has been focused on this topic in TD individuals.

### 1.3. Relationships in the Processing of Pitch between Music and Prosody in Typically Developing Individuals

Conclusions as to whether music and prosody share processing mechanisms in TD individuals have been drawn from studies in which different research strategies have been used. Results showing that music skills predict performance on prosody tasks have been taken as evidence supporting the existence of relationships between the two domains [15,16,17]. Music skills related to pitch processing (e.g., pitch direction judgment or tonal memory) have been reported to be the best predictors of performance on pitch-related prosody tasks (e.g., intonation analysis) in the native language of TD adults [15]. Delogu and collaborators studied this relationship in a foreign tonal language [16,17]. The results showed that, in both TD adults and TD children, higher skills for melodic discrimination were associated with better performance on a prosody task of lexical tone discrimination. These results were explained by the authors to be a consequence of music-to-language transfer effects and to show common cognitive processing mechanisms for pitch processing in the two domains.

Shared mechanisms have also been found in studies on the neural underpinnings of pitch processing in music and prosody. Thus, in TD adults, equivalent strong pitch variations in both music and prosody elicit large positive components that are bilaterally distributed over parieto-temporal sites [18,19]. Similar results have been found in TD children [20].

Research comparing musically trained and untrained TD individuals has also provided data supporting the existence of relationships between music and prosody. Musically trained TD adults and children outperform musically untrained peers in detecting subtle pitch variations, not only in music, but also in the prosodic contours of their native language [19,20,21]. Musically trained TD adults also perform better than musically untrained TD individuals on prosody tasks in a foreign language when they are presented with either prosodic contours or lexical tones [17,22,23,24,25,26]. Furthermore, musical training not only facilitates performance on pitch-related music and prosody tasks, but also enhances pitch processing at both the cortical and the subcortical levels [13,18,19,20,23,24,26]. These transfer effects would thus contribute to showing that music and prosody share cognitive and neural mechanisms for pitch processing in TD individuals (e.g., [18,19,20,27]).

### 1.4. Aims and Hypotheses of the Study

As mentioned above, to our knowledge, no previous studies have evaluated whether, in WS, pitch is processed in the domains of music and prosody through common cognitive mechanisms, as observed in typical development. Considering the relevance of the results of such a study for the previously discussed debates on modularity and the cognitive architecture of individuals with WS, this research aimed to fill this gap. Following prior work on TD individuals (e.g., [15,16,17]), we administered pitch-related music and prosody tasks to children with WS and their TD peers and studied the relationships between the participants’ performance on these tasks. We also evaluated whether the participants’ music skills could predict their results on the prosody tasks. It was hypothesized that, in WS, the processing of pitch in music and prosody is not independent, but subserved by common mechanisms, as observed in typical development.

## 2. Experimental Section

### 2.1. Participants

Fourteen children with WS participated in the study. All participants with WS had the clinical features of the WS phenotype (e.g., [36]). They also had a positive FISH (fluorescent *in situ* hybridization) test to confirm the gene deletion and WS diagnosis. As previously mentioned, the pitch-related music and prosody skills of individuals with WS are lower than those of TD individuals of the same chronological age [45,59,62,66]. Thus, we expected the performance level of individuals with WS in pitch-related music and prosody tasks to be lower than that of TD individuals. To check whether the music and prosody skills related to the pitch processing of participants with WS were at the level that is typically reported in the literature, a control group of 26 TD children matched on chronological age was also included in the study. Moreover, the inclusion of this group allowed us to compare the extent of the music-prosody relationships between the WS participants and their TD peers. A control group of TD children matched on chronological age was preferred over a group matched on mental age because matching on mental age would involve having TD participants who are younger than the participants with WS. In turn, the differences in life experience that such a difference in chronological age would bring could bias the results of the study (e.g., [44,45]). No significant differences were found between the WS and TD groups for chronological age (*p* = 0.98). However, as expected, full-scale IQ, verbal IQ and performance IQ, as measured with the Wechsler Intelligence Scale for Children-IV (WISC-IV) battery [67], were significantly lower in the WS group than in the TD group (*p* < 0.001 for all comparisons). The mean and range of intelligence measurements in the WS group were consistent with data reported in the literature in this respect (e.g., [68]). The descriptive characteristics of the WS and TD groups are shown in Table 1.

**Table 1:** Descriptive characteristics of the Williams syndrome (WS) and typically developing (TD) groups. Values presented in parentheses represent standard deviations.

Descriptive Characteristics	WS group	TD group
*N*	14	26
Gender (M/F)	7/7	14/12
Mean chronological age	13.58 (2.65)	13.55 (2.66)
Chronological age range	8.42–16.83	8.00–16.92
Full-scale IQ	49.29 (5.9)	118.23 (10.58)
Verbal IQ	61.50 (9.43)	117.27 (11.05)
Performance IQ	51.50 (7.28)	109.12 (10.89)

Although the sample size of the WS group was as large as or even larger than that used in prior studies on the music, prosody or other cognitive skills of individuals with WS (e.g., [29,30,40,45,63,66,69,70,71]), the number of TD participants was enlarged to increase the power of the study [72]. As reported by their parents, no participants suffered from hearing or visual impairment or had any other clinical diagnoses. No participant had received prior musical training. The participants with WS were recruited through a national Williams syndrome association, and the TD children were recruited through mainstream schools.

### 2.2. Materials and Procedure

A musical pitch discrimination task was designed for this study. The participants were presented with pairs of tones and asked to discriminate whether the tones were the same or different. A discrimination task was preferred over a task in which the participants had to judge pitch direction (*i.e.*, to determine whether a pitch was higher or lower than another pitch) because children with WS may have difficulty understanding the concept of pitch height [29]. Thus, discrimination tasks have been successfully used as the methodological choice in prior studies on the music skills of individuals with WS [45,50]. The tones were played with a tuned Samick piano and digitally recorded (a sampling frequency of 22.05 KHz) with a laptop (HP, Intel Pentium M Processor 1.60 GHz 800 MHz, SoundMAX Integrated Digital Audio sound card). Each tone lasted for 1 s and had an intensity of 70 dB, as checked and modified, if necessary, with PRAAT [73]. The presented tones corresponded to notes from the Western equal-tempered scale (A_4_ = 440), ranging from C#_4_ to C_5_. Within each pair, tones were separated by a 1-s silence. The task was composed of 20 items: two examples, two practice items and 16 experimental items. Half of the pairs were the same, and half of them were different. When the pairs were different, the following intervals were formed: minor second, major second, minor third, major third, perfect fourth, perfect fifth, major sixth and major seventh. Half of these intervals were ascending, and half of them were descending.

The short-item discrimination and long-item discrimination tasks of the Spanish Profiling Elements of Prosody in Speech-Communication (PEPS-C) battery [74] were also administered to all the participants. These tasks have been previously successfully used for the assessment of prosodic skills in individuals with WS [62,63,66]. In both tasks, the participants had to discriminate whether pairs of prosodic contours were the same or different. Specifically, the pairs of prosodic contours were the laryngograph recordings of minimal pairs of verbal items (initially spoken in Spanish, the native language of all the participants) in which the meaning was only distinguished by prosody. Since laryngograph recordings show the laryngeal signal of words, they do not include segmental information, but maintain the original prosodic contours [75]. In the short-item discrimination task, differences between prosodic contours depended on changes in the parameter of pitch. For example, a minimal pair formed by the word “cake” (*tarta*, in Spanish) with interrogative (rise contour) and with declarative intonation (fall contour) would constitute different items. Instead, in the long-item discrimination task, not only pitch, but also loudness and mainly length distinguished pairs of prosodic contours. For example, a minimal pair with the words “pink and black and green socks” (*calcetines rosas y negros y verdes*, in Spanish), where the aforementioned prosodic parameters distinguished whether the bi-colored socks are pink and black or black and green, would form an item. The length of the prosodic contours (short or relatively long contours) also distinguished the two tasks. Thus, within each pair, each stimulus lasted between 0.38 and 1.51 s (2 to 4 syllables, 1 word) in the short-item discrimination task and between 1.40 and 1.98 s (7 to 10 syllables, 3 or 4 words) in the long-item discrimination task. The structure of the two prosody tasks was the same as that in the musical pitch discrimination task. Thus, two examples, two practice items and 16 experimental items were presented. Likewise, half of the items were the same, and half of them were different.

For the three discrimination tasks (*i.e.*, musical pitch discrimination task, short-item discrimination task and long-item discrimination task), responses on different items were classified as hits if the participants answered “different”, and responses on same items were classified as false alarms if the participants answered “different”. Following prior literature (e.g., [42,49]), a discrimination score was obtained for each participant by subtracting the percentage of false alarms from the percentage of hits; thus, the maximum score for this measurement was 100. To account for both sensitivity to perceiving different items and possible answer biases [76], the discrimination score was used as the dependent variable for the analyses.

All the participants were individually assessed. Participants with WS were assessed in a quiet room of their residences, and TD participants were evaluated in a quiet room in their education centers. The music and prosody tasks were presented to the participants via the speakers of a laptop (Hewlett-Packard, Palo Alto, CA, USA; Intel Pentium M Processor 1.60 GHz 800 MHz, SoundMAX Integrated Digital Audio sound card) at a comfortable listening level. In both the WS and the TD groups, the parents of participants gave their written informed consent for their children to participate in the study. The study had been previously approved by the review board of the university.

## 3. Results

### 3.1. Inter-Group Differences in the Music and Prosody Tasks

Discrimination scores obtained in the musical pitch discrimination, short-item discrimination and long-item discrimination tasks for each group are presented in Table 2. The percentages of total correct answers are also shown in the table. Prior to the analysis of the discrimination scores, the latter variable was used to assess whether the task performance was significantly above chance in the WS group. Thus, one-sample *t*-tests were conducted on the percentage of total correct answers for each task with the chance level set at 50. The performance of the WS group was significantly above chance in the three tasks (musical pitch discrimination task: *t*(13) = 7.54, *p* < 0.001, *r* = 0.90; short-item discrimination task: *t*(13) = 6.40, *p* < 0.001, *r* = 0.87; long-item discrimination task: *t*(13) = 5.63, *p* < 0.001, *r* = 0.84). Near-to-ceiling scores were found for the musical pitch discrimination and short-item discrimination tasks in the TD group.

**Table 2:** Mean discrimination scores and percentages of total correct answers obtained in the music and prosody tasks used in the study. Values presented in parentheses represent standard deviations.

Task and Score	WS group	TD group
*Musical Pitch Discrimination Task*		
Discrimination score	69.64 (34.57)	93.27 (11.85)
Percentage of total correct answers	84.82 (17.28)	96.63 (5.92)
*Short-item Discrimination Task*		
Discrimination score	58.93 (34.47)	94.23 (10.14)
Percentage of total correct answers	79.46 (17.24)	97.12 (5.07)
*Long-item Discrimination Task*		
Discrimination score	42.86 (28.47)	86.54 (12.71)
Percentage of total correct answers	71.43 (14.23)	93.03 (6.21)

Discrimination scores were inspected for outliers. One outlier was found in the WS group for the long-item discrimination task. In the TD group, in both the musical pitch discrimination and the short-item discrimination tasks, another outlier (the same participant in the two tasks) was found. As shown in Table 2, the discrimination scores for the WS group were lower than those for the TD group. To assess whether the differences between the groups were significant, independent *t-*tests were conducted for each task. When all the data were included in the analyses, as expected, the WS group performed worse than the TD group on the three tasks (musical pitch discrimination task: *t*(38) = −2.48, *p* = 0.026, *r* = 0.37; short-item discrimination task: *t*(38) = −3.75, *p* = 0.002, *r* = 0.52; long-item discrimination task: *t*(38) = −5.46, *p* < 0.001, *r* = 0.66). The results did not differ when the outliers were excluded from the analyses.

### 3.2. Music and Prosody Relationships for Pitch Processing

To study the relationships between the music and prosody tasks, we first calculated bivariate correlations between the musical pitch discrimination task and the short-item and long-item discrimination tasks for each group separately. With the outliers included in the analyses, in the WS and TD groups, there was a significant relationship between the musical pitch discrimination task and the short-item discrimination task, *r* = 0.77, *p* = 0.001; *r* = 0.60, *p* = 0.001, respectively. However, neither in the WS group nor in the TD group was there a significant relationship between the musical pitch discrimination task and the long-item discrimination task (*p* > 0.05). The two prosody tasks were not correlated for either the WS group or the TD group (*p* > 0.05). When the outliers were excluded, the results from the correlation analyses remained the same, except for the relationship found for the TD group between the musical pitch discrimination and short-item discrimination tasks. Thus, once the outlier was removed, in the TD group, no significant correlation was obtained between these two tasks (*p* > 0.05).

We also conducted stepwise regression analyses to test whether the skills for discriminating musical pitch could predict performance on the two prosody tasks. Both prosody and music are related to auditory memory, and thus, the possible predicting effect of musical pitch discrimination on prosody may actually be explained by auditory memory (e.g., [27]). To control for this possibility, not only the musical pitch discrimination scores, but also the scores obtained in the forward digit span subtest of the WISC-IV were introduced into the stepwise regression analyses. Furthermore, to test whether, rather than musical pitch discrimination, other linguistic skills are better predictors of prosody skills, scores on the vocabulary subtest of the WISC-IV were also considered in the analyses. Table 3 shows the means and standard deviations for the forward digit span subtest and the vocabulary subtests of the WISC-IV for both the WS and the TD groups. The participants with WS performed significantly worse than their TD peers in both tasks (forward digit span: *t*(38) = −6.00, *p* < 0.001, *r* = 0.70; vocabulary: *t*(38) = −13.19, *p* < 0.001, *r* = 0.91). Additionally, taking into account that prosody skills change with age during the age range of the sample included in this study [74,77], chronological age was also included as a potential predictor.

**Table 3:** Mean discrimination scores (SD) on the forward digit span and the vocabulary subtests of the Wechsler Intelligence Scale for Children-IV (WISC-IV). Values presented in parentheses represent standard deviations.

Subtest	WS group	TD group
Forward digit span (maximum score = 16)	5.5 (1.45)	9.27 (7.03)
Vocabulary (maximum score = 68)	20.64 (5.80)	49.65 (7.03)

**Figure 1:**
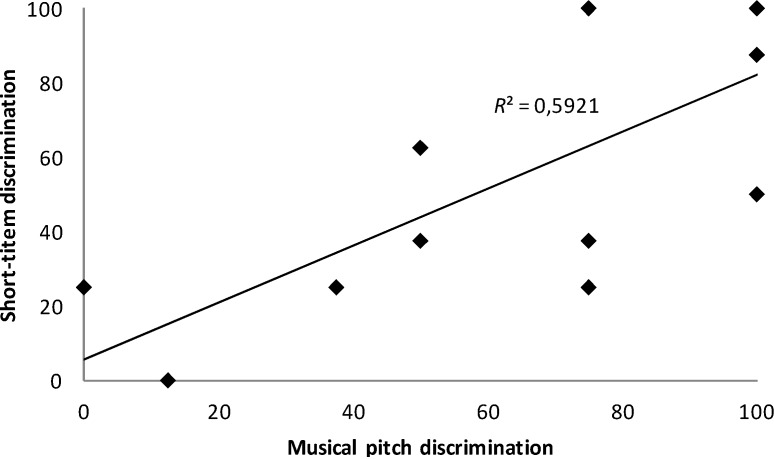
Relationships between musical pitch discrimination and performance on the short-item discrimination task in the WS group.

For the WS group, when we introduced musical pitch discrimination, forward digit span, vocabulary and chronological age as independent variables and scores on the short-item discrimination task as the dependent variable in the stepwise regression analysis, only musical pitch discrimination significantly predicted performance on the short-item discrimination task, *F*(1,12) = 17.42, *p* = 0.001, *R*^2^ = 0.59. This relationship is shown in Figure 1. When the same analysis was conducted with scores on the long-item discrimination task as the dependent variable, no significant predictors were found (*p* > 0.05). The same results were obtained when the outlier was removed from the analyses.

**Figure 2:**
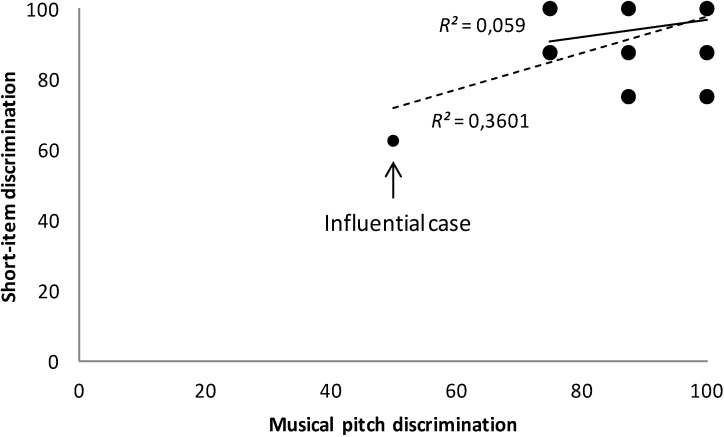
Relationships between musical pitch discrimination and performance on the short-item discrimination task in the TD group before and after removing an influential case. The dashed line represents the regression function before the removal of the influential case. The solid line shows the regression function after the removal of this case.

For the TD group, when considering all the participants, performance on the short-item discrimination task was significantly predicted only by musical pitch discrimination, *F*(1,24) = 13.50, *p* = 0.001, *R*^2^ = 0.36. Nevertheless, Cook’s distance revealed that one case was exerting undue influence over the parameters of the model (Cook’s D = 2.05), *i.e.*, the outlier. When this case was removed and the stepwise regression analysis was conducted again, none of the independent variables showed statistical significance (*p* > 0.05). Figure 2 shows the results for the two regression analyses. As for the long-item discrimination task, the stepwise regression analysis showed that, unlike in the WS group, where no significant predictors were found, in the TD group, scores on the forward digit span subtest did reach significance, *F*(1,24) = 5.27, *p* = 0.03, *R*^2^ = 0.18.

## 4. Discussion

This research aimed to assess whether children with WS process pitch in both music and prosody through common cognitive mechanisms, *i.e.*, it sought to study the relationships between music and prosody for pitch processing in children with WS. Before delving into these relationships, we first assessed whether the pitch-related music and prosody skills of the children with WS were at the level that is typically reported for individuals with WS. An initial analysis showed that the task performance of the children with WS was significantly above chance. As expected, the participants with WS performed significantly worse than their TD peers matched on chronological age on both the music task and the two prosody tasks. Thus, the results for the musical pitch discrimination, short-item discrimination and long-item discrimination tasks were consistent with those previously reported in the literature on the topic [45,59,62,66].

Regarding the relationships between the music and prosody tasks used in the study, in the WS group, a significant correlation was found between the scores obtained on the musical pitch discrimination task and the scores obtained on the short-item discrimination task. The stepwise regression analysis showed that musical pitch discrimination skills predicted performance on the short-item discrimination task even when other potentially explanatory variables (*i.e.*, chronological age, auditory memory and vocabulary level) had been factored out. As in prior studies with TD individuals in which similar findings (e.g., the predictive effect of music skills on prosody performance) have been interpreted as evidence supporting the existence of common cognitive mechanisms for pitch processing in music and prosody [15,16,17], our results could also be taken as evidence that children with WS process pitch in both music and prosody through common mechanisms. In turn, the aforementioned results would provide evidence against the idea that WS illustrates the modularity of both music and language [29,30,31,40], at least with respect to the linguistic component of prosody. Thus, if music and prosody were independent modules, domain-specificity would be expected. Instead, in light of the results of the current study, in individuals with WS, the cognitive mechanisms that are responsible for pitch processing seem to be not restricted to music, but to be involved in the domain of both music and prosody. The current view of the architecture of music processing claims that the music module is formed by distinct music-specific modules [8]. Nevertheless, within the same view, the so-called contour analysis component, which is responsible for abstracting pitch trajectories, is thought to operate not only in music, but also in prosody [8]. Similarly, from our results, we would conclude that, in the cognitive architecture of individuals with WS, pitch processing is not specific to music, but shared with prosody.

Although a significant relationship was found between the musical pitch discrimination task and the short-item discrimination task in individuals with WS, it should be noted that, in the same participants with WS, no significant correlation was found between the musical pitch discrimination task and the long-item discrimination task, and none of the possible predictors that were included in the stepwise regression analysis contributed in explaining the variance in performance on this prosody task. The difference between the short-item discrimination and the long-item discrimination tasks in terms of their relationships with skills for musical pitch discrimination could be accounted for by considering the different parameters that are involved in the tasks. As previously mentioned, while pitch is the main parameter distinguishing the prosodic contours that are included in the short-item discrimination task, not only pitch, but also loudness and, mainly, length characterize the prosodic contours of the long-item discrimination task. In turn, pitch is the sole parameter distinguishing the musical notes of the musical pitch discrimination task. Thus, only for the musical discrimination and short-item discrimination tasks is pitch the most informative parameter. Therefore, the fact that, unlike short-item discrimination, the long-item discrimination task mainly requires the processing of length apart from the processing of pitch and loudness would explain why musical pitch discrimination skills did not predict performance on this prosody task. The difference in the parameters that are involved in the two prosody tasks would also account for the lack of a significant correlation between them. As previously discussed, the relationships found between the musical pitch discrimination and the short-item discrimination tasks in individuals with WS suggest that children with WS process pitch in music and prosody through the same cognitive mechanisms. Loudness and length are also important parameters of both music and prosody [9,10,11,12]. In this study, we focused on the processing of pitch and, therefore, we cannot draw any conclusions regarding the modularity or the relationships in the processing of loudness and length in music and prosody. Further research should tackle this issue.

Individuals with WS have an intellectual disability (e.g., [59]). Thus, it might be argued that the significant relationships found in the WS participants between the musical pitch discrimination and short-term discrimination tasks may only result from their cognitive impairments or that, considering the worse performance of the WS group compared to the TD group on the three tasks of the study, it is not possible to disentangle the effects of the general cognitive deficits of individuals with WS from the specific factors that were analyzed in this research (*i.e.*, the relationships for the processing of pitch between music and prosody). However, it should be noted that the predictive effect of musical pitch discrimination on the short-item discrimination task was found even after we controlled for the effect of other important variables that are indicative of the cognitive impairment of individuals with WS (*i.e.*, vocabulary and auditory memory). Moreover, if the intellectual disability of individuals with WS were the only factor explaining the results of the study, no differences would have been found in the relationships between the musical pitch discrimination task and the two different prosody tasks. Instead, we found that musical pitch discrimination was a significant predictor of the short-item discrimination task (*i.e.*, the prosody task based on pitch changes) only. These results show that the relationships found in individuals with WS between the musical pitch discrimination and short-item discrimination tasks cannot be accounted for by the general cognitive impairment of individuals with WS.

As observed in the WS group, in the TD group, no significant correlations were found between the musical pitch discrimination and the long-item discrimination tasks or between the short-item discrimination and long-item discrimination tasks. However, in the TD group, the musical pitch discrimination task was not significantly correlated with the short-item discrimination task when an outlier was removed from the analyses. Similarly, once the influential case was removed, musical pitch discrimination did not predict performance on the short-item discrimination task. Moreover, unlike in the WS group, in the TD group, auditory memory (e.g., forward digit span) did not predict performance on the long-item discrimination task.

Rather than suggesting the existence of independent processing mechanisms, the lack of a significant relationship between the musical pitch discrimination and the short-item discrimination task for the TD participants could be explained by the near-to-ceiling effects found for these tasks. Thus, before we removed the outlier (*i.e.*, a participant who obtained relatively lower scores in comparison with the rest of the group), a significant correlation between the musical pitch discrimination task and the short-item discrimination task was found. Likewise, prior to the removal of the outlier, musical pitch discrimination was found to be a significant predictor of performance on the short-item discrimination task. The near-to-ceiling effects found for the musical pitch discrimination and short-item discrimination tasks make it impossible to interpret the results obtained for these tasks. As explained in Section 2.2, we used a musical pitch discrimination task and not a pitch direction judgment task to avoid possible confounding effects linked to the difficulties that children with WS have for understanding the concept of pitch height. Although this procedure ensured that the participants with WS understood the task (as shown by their significantly above chance level performance), it also made the musical pitch discrimination task too easy for the TD group. For the short-item discrimination task, ceiling scores have been found starting from the age of 13 years in TD children [74]. Considering the associations found in prior studies between pitch-related music and prosody tasks in TD individuals (e.g., [15,16,17,18]), future research on TD children who are younger than those included in the current study should elucidate the relationships between the musical pitch discrimination and short-item discrimination tasks. The use of a different pitch-related musical task (e.g., determining whether one pitch is higher or lower than another) may also help to clarify the issue.

With regard to the long-item discrimination task, as mentioned above, in the TD group, auditory memory significantly predicted performance on this task. Nevertheless, this variable did not account for any of the variance in the short-item discrimination task. As their names indicate, the short-item discrimination and long-item discrimination tasks include short and relatively long prosodic contours, respectively. Therefore, the memory load of the long-item discrimination task is higher than that of the short-item discrimination task. This could explain why auditory memory was a significant predictor only for the long-item discrimination task in the TD group. In the WS group, no relationship was found between the forward digit span task and the long-item discrimination task. Specific difficulties associated with auditory memory in individuals with WS (e.g., [78]) may contribute to accounting for this result.

As for the remaining potential predictors, no significant effects were found for chronological age or vocabulary level in any of the regression analyses. The lack of a significant relationship between chronological age and performance on the prosody tasks in the TD group may be explained by considering that the prosody skills that were assessed in this study are already acquired by 11–13 years of age [74], and the present research included participants who were older than this age range. It should also be noted that chronological age usually is not a good predictor of performance in developmental disorders, such as WS [79]. With respect to vocabulary level, although it may be considered surprising that no relationships were found with any of the prosody tasks, these results are consistent with data reported in the literature on this topic in both TD individuals and children with WS [66].

An important finding of the current study is the significant relationship found for participants with WS between performance on the musical pitch discrimination task and that on the prosody short-item discrimination task. As previously mentioned, this result suggests that, in individuals with WS, pitch is processed through shared mechanisms in music and prosody. Although, as mentioned above, the same has been reported for TD individuals in prior studies (e.g., [15,16,17,18,19,20,27]), our findings on individuals with WS should not be taken as further evidence regarding the cognitive organization of pitch processing in TD individuals [51]. Thus, it is important to note that although common mechanisms subserve pitch processing in music and prosody in both children with WS and TD individuals, the processes themselves are not necessarily the same in both groups. In fact, the difference in the predicting effect of auditory memory on the scores on the long-item discrimination task between the WS and the TD groups in this study suggests that atypical processes may underlie prosody performance in individuals with WS. In line with this, it should be considered that, compared to TD individuals, those with WS have been reported to have different neural processes underlying behavior in prosody tasks [80]. Different cognitive and neural processes have also been found for the domain of music in WS [42,61,81,82].

As previously mentioned, in the current study, skills for musical pitch discrimination predicted performance on the short-item discrimination task for children with WS. As explained in Section 2.2, this prosody task assesses the ability to discriminate pairs of prosodic contours that are based on pitch [74,75]. Therefore, it evaluates the perception of this parameter, but does not test the comprehension of the linguistic functions that such a parameter can express. Thus, pitch plays an important role in different communicative functions of language, such as establishing conversational turns, providing cues for segmenting the speech-chain, expressing the focus of an utterance or providing lexical information (e.g., [83,84,85,86,87]). In TD individuals, music skills are related to not only the perception of pitch in prosody, but also the comprehension of its lexical communicative function (e.g., [16,17,26,88]). Further research should evaluate whether music skills are related to the understanding of the communicative functions expressed by pitch in individuals with WS, as well.

Our finding that children with WS process pitch in music and prosody through common cognitive mechanisms opens up new paths for intervention and educational programs. As mentioned in Section 1.3, musical training enhances the prosody skills of TD children and adults (e.g., [18,19,20,21]). Taking into account that TD individuals process music and prosody through common mechanisms, the positive effect of musical training is explained by the fact that such training enhances the sensitivity to the acoustic parameters that are involved in speech prosody [27]. Individuals with WS have deficits for the perception and comprehension of the prosodic parameters and their communicative functions [62,63,66]. Thus, if, as found in the current research, pitch-related music and prosody skills are subserved by shared cognitive processing mechanisms in individuals with WS, then musical training may also improve their prosody skills. Individuals with WS usually have a high interest in music and are motivated to participate in musical activities [89,90]. Therefore, in combination with speech and language therapy, musical training may be a suitable intervention tool. Future studies should evaluate whether musical training can improve the prosody skills of individuals with WS.

## 5. Conclusions

Studies on WS have been a breeding ground for debates on the modularity of mind (e.g., [28,31,32]). Despite the existing controversy regarding the modularity of language and music in WS [29,30,31,40,41,49], this is the first work focused on the possible relationships between the processing mechanisms for music and the language component of prosody in this population. In the current research, the skills of children with WS for musical pitch discrimination were related to their ability to discriminate prosodic contours based on pitch. This result suggests that children with WS process pitch in both music and prosody through common cognitive mechanisms. Therefore, this study provides evidence against the view that music and language (at least with respect to the component of prosody) constitute independent modules in WS. The reported relationships between music and prosody for pitch processing in individuals with WS are consistent with previous findings in TD individuals [15,16,17]. Nevertheless, these results should not be taken as evidence that the processes underlying music and prosody are the same between individuals with WS and TD individuals. Thus, the differences found between the WS group and the TD group with respect to the relationships between auditory memory and the long-item discrimination task suggest that atypical processes subserve prosodic performance in WS.

In addition to the theoretical implications, the results of this study may also have practical implications. In TD individuals, shared mechanisms for the processing of pitch in music and prosody account for the facilitating effect of musical training on prosodic performance [19,20,21]. As explained above, our results highlight common mechanisms for the processing of pitch in music and prosody in WS. Therefore, musical training may also enhance prosodic performance in individuals with WS. More research is needed to clarify whether musical training could be used as an intervention tool to improve the prosody skills of individuals with WS.
